# Clarifying the novelty of TLR4-targeted therapy in lung ischemia-reperfusion injury

**DOI:** 10.1016/j.jhlto.2026.100700

**Published:** 2026-09-26

**Authors:** Akira Shimamoto

**Affiliations:** Department of Medical Sciences and Nursing Innovation, University of Kochi Graduate School of Nursing, Kochi, Japan

To the Editor:

I read with great interest the article by Goda et al., “Pharmacological inhibition of toll-like receptor 4 suppresses ischemia-reperfusion injury-induced inflammation and improves lung allograft function after transplantation.”^1^ The authors demonstrated that administration of the Toll-like receptor 4 (TLR4)-inhibitory peptide VIPER reduced pulmonary edema and inflammatory responses and improved early graft function in a murine orthotopic lung transplantation model. In particular, the use of 18-hour cold preservation followed by orthotopic lung transplantation represents a clinically relevant extension of experimental studies of lung ischemia-reperfusion injury (LIRI).^1^

However, I believe that the historical development of TLR4-targeted strategies in LIRI warrants clarification when interpreting the novelty of this study.

Nearly two decades ago, we demonstrated that TLR4 is a critical mediator of LIRI using TLR4-deficient mice.^2^ Compared with wild-type mice, TLR4-deficient animals exhibited marked reductions in vascular permeability, pulmonary myeloperoxidase activity, leukocyte accumulation, and inflammatory cytokine and chemokine production following lung ischemia and reperfusion. We further demonstrated attenuation of downstream inflammatory signaling, including mitogen-activated protein kinase activation and nuclear translocation of NF-κB and activator protein-1.^2^ These findings established TLR4 as an upstream mediator of the inflammatory response in LIRI and identified TLR4 signaling as a potential therapeutic target.

Importantly, our subsequent work extended these mechanistic observations to pharmacological intervention. In 2009, we reported at the International Society for Heart and Lung Transplantation that pretreatment with the TLR4 antagonist eritoran significantly attenuated NF-κB activation, vascular permeability, myeloperoxidase activity, and inflammatory responses in experimental LIRI.^3^ These findings provided early evidence that direct pharmacological inhibition of TLR4 could mitigate lung ischemia-reperfusion injury.

In the same period, Zanotti et al. independently demonstrated that pharmacological inhibition of TLR4 with CRX-526 reduced ischemia-reperfusion-induced pulmonary edema in vivo and protected pulmonary microvascular endothelial cells during simulated ischemia-reperfusion.^4^ Thus, both the mechanistic importance of TLR4 and the therapeutic potential of pharmacological TLR4 inhibition in LIRI had been demonstrated before the current study.

Of further relevance, Takahashi et al. subsequently showed that pharmacological inhibition of TLR4 signaling ameliorated LIRI under acute hyperglycemic conditions.^5^ This study is appropriately cited by Goda et al. However, the earlier investigations that established the mechanistic role of TLR4 in LIRI and demonstrated the feasibility of direct pharmacological TLR4 inhibition are not discussed in the current report.

Accordingly, I suggest that the principal novelty of the study by Goda et al. lies not in identifying TLR4 as a therapeutic target for LIRI per se, nor in demonstrating the feasibility of pharmacological TLR4 inhibition, but rather in extending this established therapeutic concept to a murine orthotopic lung transplantation model following prolonged cold preservation and demonstrating the efficacy of the TLR4-inhibitory peptide VIPER in that setting.

This distinction does not diminish the significance of their findings. On the contrary, it more precisely places their study within the progression of research from mechanistic identification of TLR4 as a mediator of LIRI, through pharmacological intervention, to application in an orthotopic transplantation model.

Accurate recognition of this progression is important not merely for attribution of priority, but also for understanding how mechanistic observations evolve into therapeutic strategies and, ultimately, into experimental models that more closely resemble clinical lung transplantation.

## Funding

The author’s ongoing research on lung ischemia-reperfusion injury and pharmacological preconditioning is supported by JSPS KAKENHI Grant Number 23K08311. No specific funding was received for the preparation of this correspondence.

## Declaration of Generative AI and AI-assisted technologies in the writing process

During the preparation of this work, the author used ChatGPT (OpenAI) to assist with language editing and manuscript organization. After using this tool, the author reviewed and edited the content as needed, verified the cited literature, and takes full responsibility for the content of the manuscript.

## Declaration of competing interest

The author declares that there are no competing interests.
